# GLUL Confers Perivascular Cancer‐Associated Fibroblasts With Pro‐Angiogenic Capacity to Promote Glioma Progression

**DOI:** 10.1002/advs.202513184

**Published:** 2025-12-08

**Authors:** Qing Zhang, Yida Liu, Zhi Zhang, Yang Wang, Fusheng Liu

**Affiliations:** ^1^ Department of Neurosurgery Beijing Chao‐Yang Hospital Capital Medical University Beijing 100020 China; ^2^ Department of Neurosurgery Beijing Tiantan Hospital Affiliated to Capital Medical University Beijing 100070 China; ^3^ Brain Tumor Research Center Beijing Neurosurgical Institute Capital Medical University Beijing 100070 China; ^4^ Beijing Laboratory of Biomedical Materials Beijing 100070 China

**Keywords:** glioblastoma, cancer‐associated fibroblasts, angiogenic capacity, GLUL, tumor microenvironment

## Abstract

Glioblastoma (GBM) is a malignant brain tumor characterized by profound angiogenic activity and immunosuppressive features. A burgeoning body of research has focused on elucidating the functional effects of stromal cells within the tumor microenvironment (TME) and developing stroma‐targeted therapeutic strategies. Notably, cancer‐associated fibroblasts (CAFs), essential stromal components of the TME, have garnered significant attention for their functional orchestration in glioma progression. The proteomic landscape of human primary CAFs from GBM samples has revealed the dynamic remodeling of differential protein expression in the TME, but the functional role of glutamate‐ammonia ligase (GLUL) as a novel CAF target remains elusive. This study confirms that GLUL knockdown profoundly abrogated the architectural intricacy inherent to CAF‐supported vasculature in vitro and in vivo. Additionally, CAF‐specific GLUL knockdown attenuates tumor growth and extends median survival in a humanized orthotopic glioma model. Furthermore, GLUL‐driven activation of PI3K/AKT signaling as the central regulator of CAF‐mediated vascular niche formation is delineated in glioma progression. This study highlights that targeting GLUL in CAFs is a novel stroma‐focused therapeutic paradigm for GBM by disrupting pro‐angiogenic signaling. Collectively, these findings elucidate key aspects of CAF biology and their regulatory functions in tumor progression, underscoring the therapeutic potential of targeting CAFs in GBM.

## Graphical abstract

Schematic illustration of the proposed model. Primary CAFs are isolated from fresh human GBM specimens according to established protocols. GLUL is essential for pro‐angiogenic capacity of CAFs through its impact on the PI3K/AKT pathway. GLUL enhances the pro‐angiogenic capacity of CAFs, driving aberrant tumor vasculature that fuels tumor growth; conversely, GLUL knockdown restores vascular normalization and suppresses tumorigenesis.

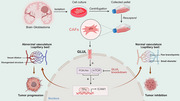

## 1 Introduction

Glioblastoma (GBM) is the most common and malignant primary intracranial tumor.^[^ ^1^, ^2^ ^]^ Dysregulated remodeling of the tumor microenvironment (TME) significantly contributes to therapeutic limitations and drug resistance, making GBM a highly challenging malignancy.^[^ ^3^, ^4^ ^]^ Despite advances in surgical intervention and various emerging therapies, the median overall survival of patients remains poor.^[^ ^5^, ^6^ ^]^ There is a compelling need to explore more effective and targeted strategies. Current clinically applied targeted drugs focus primarily on tumor cells,^[^ ^7^, ^8^, ^9^ ^]^ with the stromal cellular components within the TME being less extensively investigated. Cancer‐associated fibroblasts (CAFs) are pivotal components of the TME, and their roles in diverse cancers, including the establishment of immunosuppression, aberrant angiogenesis, extracellular matrix (ECM) remodeling, and metabolic remodeling, have spurred intensifying research efforts.^[^ ^10^, ^11^, ^12^ ^]^ CAFs further contribute to tumor progression and modulate treatment responses by secreting a diverse array of cytokines, chemokines, growth factors, and exosomes.^[^ ^13^, ^14^ ^]^ In‐depth exploration of CAF‐related mechanisms offers promising new insights for cancer therapy.

CAFs have been established as mediators of angiogenesis in multiple solid tumors. CAFs critically regulate angiogenesis through the secretion of factors such as CHI3L1, PDGFC, SDF1, WNT2 and WNT5a.^[^ ^15^, ^16^, ^17^ ^]^ In breast cancer, FOSL2 in CAFs promotes tumor progression by inducing angiogenic activity in HUVECs through the activation of FZD5/NF‐κB/ERK signaling.^[^ ^18^ ^]^ In colorectal cancer, SULF1 modulates tumor angiogenesis by promoting the angiogenic capacity of endothelial cells through VEGFA secretion from CAFs,^[^ ^19^ ^]^ and CAF‐derived microRNA‐135b‐5p was confirmed to promote cancer cell growth and angiogenesis.^[^ ^20^ ^]^ In addition, ATF4, a key regulator of the integrated stress response, remodels the ECM, further drives tumor vascularization, and fosters malignant progression and metastasis.^[^ ^21^ ^]^ However, whether primary CAFs have the potential to form vascular‐like structures and contribute to glioma vascularization remains to be elucidated. Recently, single‐cell sequencing technologies have greatly facilitated the dissection of CAF heterogeneity and subsets.^[^ ^22^, ^23^, ^24^ ^]^ Certain CAF subpopulations have also been reported to exhibit antitumorigenic functions.^[^ ^25^ ^]^ Primary contributors to CAF heterogeneity include their diverse cellular origins, dynamic phenotypic switching of individual CAF populations between tumor‐promoting or tumor‐suppressing states driven by the complex TME composition, and the existence of a diverse array of CAF subtypes with distinct functional properties.^[^ ^26^, ^27^, ^28^ ^]^

Glutamate‐ammonia ligase (GLUL), also known as glutamine synthetase, is a key enzyme in nitrogen metabolism. GLUL catalyzes the ATP‐dependent synthesis of glutamine from glutamate and ammonia. Mutations or dysregulation of GLUL are associated with various cancers.^[^ ^29^, ^30^ ^]^ Previous studies have shown that GLUL‐mediated glutamine synthesis is critical for tumor growth because it regulates amino acid transport and nitrogen metabolism, including nucleotide synthesis and ammonia detoxification.^[^ ^31^, ^32^ ^]^ An increasing number of studies on how GLUL participates in regulating cancer progression have been reported. PHF8 is recruited by c‐MYC to the promoter region of TEA domain transcription factor 1 to transcriptionally upregulate GLUL, further promoting lipid accumulation and tumor progression in renal cell carcinoma.^[^ ^33^ ^]^ YAP1 directly binds to the transcriptional start site of the GLUL promoter, increasing GLUL expression. Consequently, YAP1 knockdown reduces GLUL expression and suppresses tumor cell growth in hepatocellular carcinoma.^[^ ^34^ ^]^ Increased GLUL expression promotes ribonucleotide and asparagine biosynthesis, as well as amino acid transport, in breast cancer cells.^[^ ^29^ ^]^ In c‐MYC‐driven malignancies, GLUL inhibition suppresses cell viability, proliferation, and tumorigenesis.^[^ ^35^ ^]^ In addition, silencing GLUL reduces the migration capacity of endothelial cells to impair vessel sprouting during vascular development.^[^ ^36^ ^]^ Overall, GLUL primarily regulates tumor growth and metastasis through its roles in nutrient metabolism and transport. However, its contribution to glioma vascularization and malignant progression remains uncharacterized.

In this study, we demonstrated that GLUL deletion substantially attenuated CAF‐mediated vascular‐like structure formation and vascular mimicry in endothelial cells. In orthotopic models, GLUL knockdown suppressed microvascular formation and tumor growth, concomitant with significantly prolonged animal survival. We further reported that GLUL knockdown inhibits PI3K/AKT signaling and intercellular adhesion molecule 1 (ICAM1) expression in CAFs. Furthermore, in vivo experiments confirmed attenuated tumor‐promoting functionality in GLUL‐depleted CAFs, which correlated with demonstrably extended median survival in mice. These findings establish an essential protumorigenic role for CAFs mediated by GLUL‐dependent mechanisms, indicating promising avenues for therapeutic intervention.

## 2 Results

### 2.1 CAFs are Involved in the Formation of Vascular Mimicry in the Glioma Microenvironment

**Figure 1:**
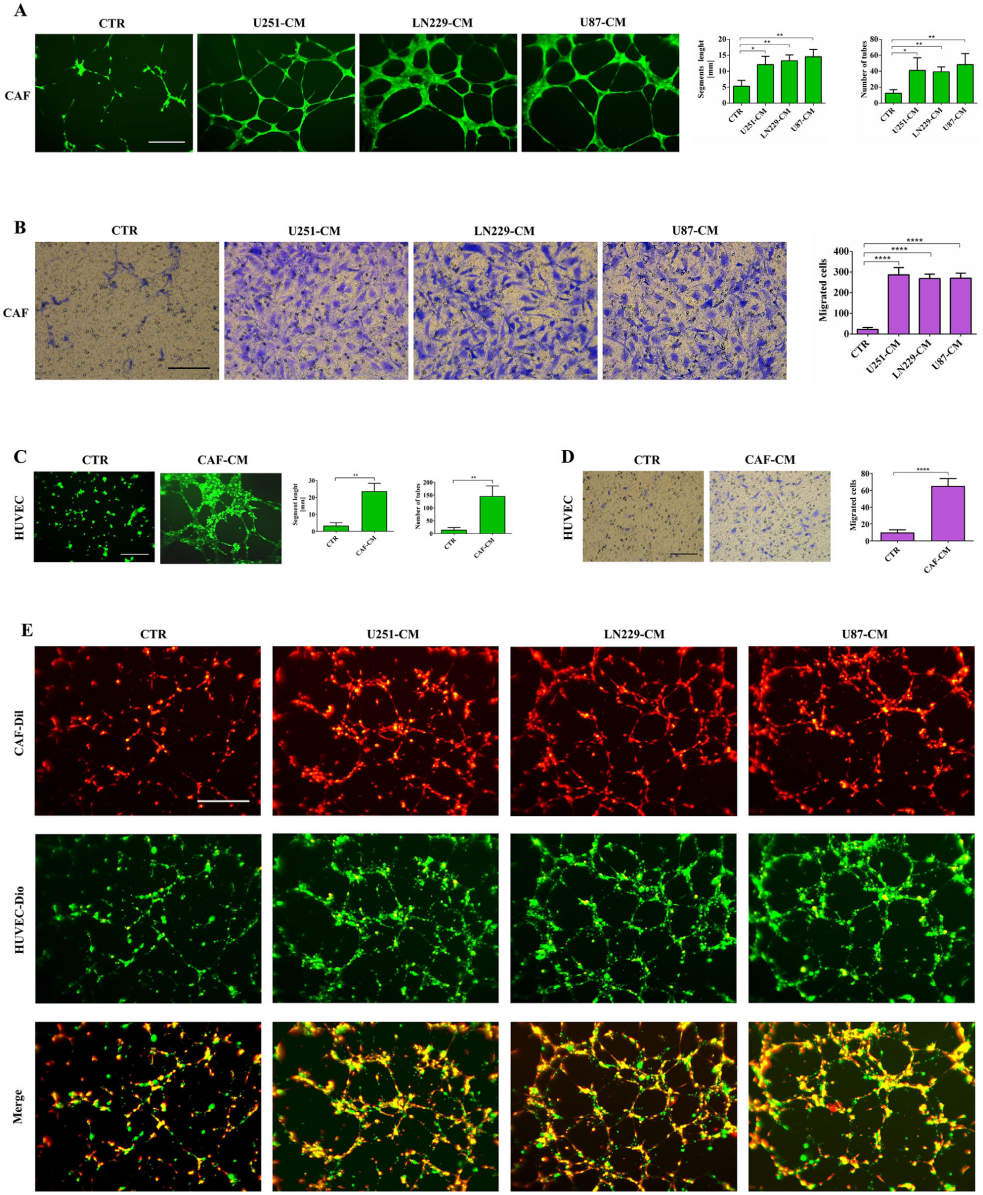
CAFs participate in glioma angiogenesis in vitro. A) Representative images of tube formation by CAFs after 8 h of incubation with CM derived from glioma cells (n = 3). Scale bar, 500 µm. ^*^ *p <* 0.05, ^**^ *p <* 0.01; one‐way ANOVA was used. B) Representative images of CAF recruitment for 24 h using CM derived from glioma patients are shown (n = 3). Scale bar, 500 µm. ^****^ *p <* 0.0001 according to one‐way ANOVA. C) Representative images of tube formation by HUVECs after 8 h of incubation with CM derived from CAFs (n = 3). Scale bar, 500 µm. ^**^ *p <* 0.01 according to Student's *t*‐test. D) Representative images of HUVEC recruitment for 24 h using CM derived from CAFs are shown. Scale bar, 500 µm. ^****^ *p <* 0.0001 according to Student's *t*‐test. E) Representative images of tube formation by CAFs and HUVECs after 8 h of incubation with CM derived from glioma (n = 3). Scale bar, 500 µm.

Our previous study revealed that fibroblasts are abundant in glioma tissues, especially in GBM.^[^ ^37^ ^]^ Our findings confirmed that the expression of FAP and a‐SMA in GBM tissues was significantly greater than that in low‐grade gliomas (Figure S1A, Supporting Information). Therefore, we employed immunofluorescence and flow cytometry to analyze the expression of CAF markers in primary cells isolated from GBM tissues (Figure S1B–E, Supporting Information), and the observed coexpression of FAP and α‐SMA was confirmed in primary fibroblasts (Figure S1F, Supporting Information), confirming the presence of CAFs in glioma tissues. We further revealed that glioma‐conditioned media (U251‐CM, LN229‐CM, and U87‐CM) significantly promoted the formation of vascular‐like structures and enhanced the migratory capacity of CAFs (**Figure** **1**A,B).

Similarly, the supernatant derived from CAFs was found to stimulate vascular mimicry and increase the motility of HUVECs (Figure 1C,D). Furthermore, within the glioma microenvironment, CAFs were observed to envelop HUVECs and cooperatively assemble into vascular structures, accompanied by a marked increase in angiogenic potential (Figure 1E). Moreover, at 24 h postvasculogenic induction, CAFs maintained vascular integrity in glioma‐CM, confirming their robust angiogenic potential (Figure S2A, Supporting Information). However, the vascular structures formed by HUVECs had almost completely appeared for 24 h (Figure S2B, Supporting Information). Therefore, we confirmed that CAFs have the potential to participate in vascular mimicry and that their powerful angiogenic capacity is significantly enhanced within the glioma microenvironment.

### 2.2 GLUL Knockdown in CAFs Attenuates Angiogenic Capacity In Vitro and In Vivo

**Figure 2:**
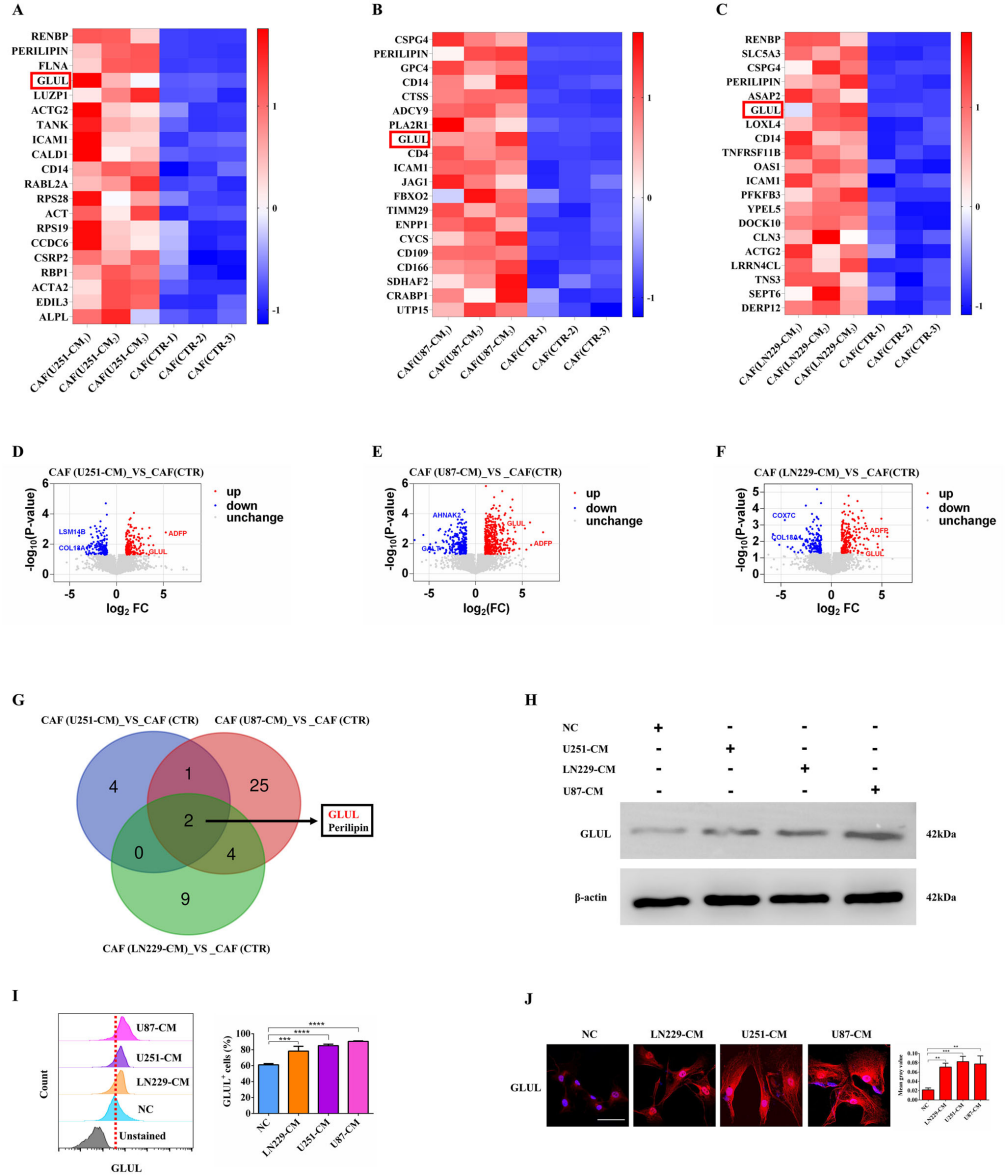
Proteomic landscapes of human primary CAFs from GBM samples revealed differential protein expression. A–C) The top 20 differentially expressed protein profiles in primary CAFs are shown in a heatmap. D–F) Volcanic maps of different proteins in primary CAFs. G. Venn diagrams showing putative targets of GLUL in CAFs. H‐J. GLUL levels were detected by western blotting, flow cytometry or immunofluorescence staining in CAFs treated with U251‐CM, LN229‐CM, or U87‐CM. The data are presented as the means ± SDs of triplicate wells. ^**^ *p <* 0.01, ^***^ *p <* 0.001, ^****^ *p <* 0.0001; one‐way ANOVA was used.

**Figure 3:**
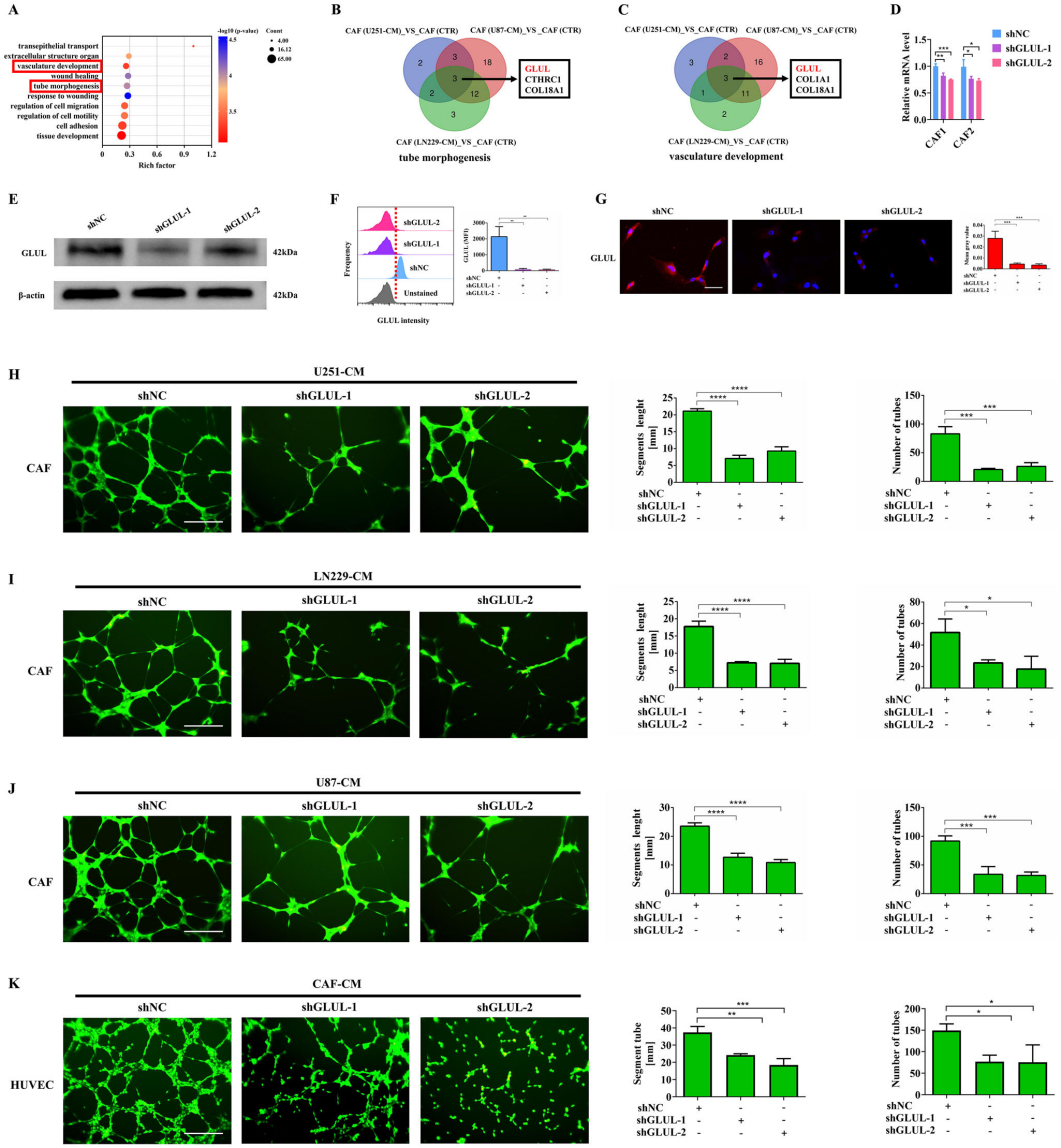
GLUL promotes the angiogenic capacity of CAFs in vitro. A) Bubble plot of Gene Ontology (GO) enrichment analysis showing significantly enriched biological processes for CAF‐related angiogenesis. The bubble size represents the number of genes enriched in the term, and the color intensity corresponds to the statistical significance (‐log10 (*P*‐value)). B,C) Venn diagrams were used to demonstrate the putative targets of GLUL in CAF‐related angiogenesis. The predicted targets from Gene Ontology were used in our analysis related to angiogenesis. D–G) GLUL levels were detected by RT‐PCR, western blotting, flow cytometry or immunofluorescence staining in the indicated cells (n = 3). Scale bar, 50 µm. ^*^ *p <* 0.05, ^**^ *p <* 0.01, ^***^ *p <* 0.001; one‐way ANOVA was used. H) Tube formation of GLUL‐knockdown CAFs for 8 h was measured after treatment with U251‐CM (n = 3). Scale bar, 500 µm. ^***^ *p <* 0.001, ^****^ *p <* 0.0001; one‐way ANOVA was used. I) Tube formation of GLUL‐knockdown CAFs for 8 h was measured after treatment with LN229‐CM (n = 3). Scale bar, 500 µm. ^*^ *p <* 0.05, ^****^ *p <* 0.0001; one‐way ANOVA was used. J) Tube formation of GLUL‐knockdown CAFs for 8 h was measured after treatment with U87‐CM (n = 3). Scale bar, 500 µm. ^***^ *p <* 0.001, ^****^ *p <* 0.0001; one‐way ANOVA was used. K. Effects of incubation with GLUL‐knockdown CAFs for 8 h on the tube formation of HUVECs (n = 3). Scale bar, 500 µm. ^*^ *p <* 0.05, ^**^ *p <* 0.01, ^***^ *p <* 0.001.

To further explore the pro‐angiogenic molecular mechanisms of CAFs in glioma, we conducted proteomic analysis of CAFs treated with CM from glioma patients. Proteomic profiling revealed distinct alterations: compared with control CM, U251‐CM induced the upregulation of 182 proteins and the downregulation of 169 proteins; U87‐CM resulted in 430 upregulated and 239 downregulated proteins; and LN229‐CM led to 210 upregulated and 163 downregulated proteins (Figure S3A, Supporting Information). Furthermore, heatmap analysis, volcano plots, and Venn diagrams collectively demonstrated that CM derived from different glioma cell lines triggered significant and distinct patterns of protein expression in CAFs, characterized by a spectrum of upregulated and downregulated proteins. (**Figure** **2**A–F; Figure S3B–F, Supporting Information). Similarly, Venn diagram analysis revealed that GLUL was a common differentially expressed protein among the three distinct treatment groups (Figure 2G). Furthermore, we employed a comprehensive in vitro validation approach integrating western blotting, flow cytometric analysis, and immunofluorescence assays to investigate the expression of GLUL (Figure 2H‐J) systematically. Subsequent integrated bubble plot visualization and Gene Ontology enrichment analysis confirmed the association of GLUL with angiogenesis‐related functional modules (**Figure** **3**A–C).

Lentivirus‐mediated RNA interference was employed to achieve targeted GLUL knockdown in CAFs. Western blotting, flow cytometric profiling and immunofluorescence coupled with RT‐qPCR analysis confirmed a marked attenuation of GLUL transcriptional output and a concomitant reduction in proteomic abundance (Figure 3D–G). Further tube formation experiments revealed that the potential of GLUL‐knockdown CAFs to participate in vascular mimicry formation was significantly diminished in different types of glioma CM, and quantitative analysis demonstrated a marked reduction in vascular segment length and the number of tubes (Figure 3H–J). Moreover, HUVECs exhibited significantly impaired vascular activity in supernatants derived from CAF‐knockdown cells compared with those derived from control cells (Figure 3K). Furthermore, we confirmed that GLUL knockdown in CAFs significantly reduced their migratory capacity (Figure S4A–C) and that supernatant from these CAF‐knockdown cells markedly inhibited HUVEC migration (Figure S4D, Supporting Information).

**Figure 4:**
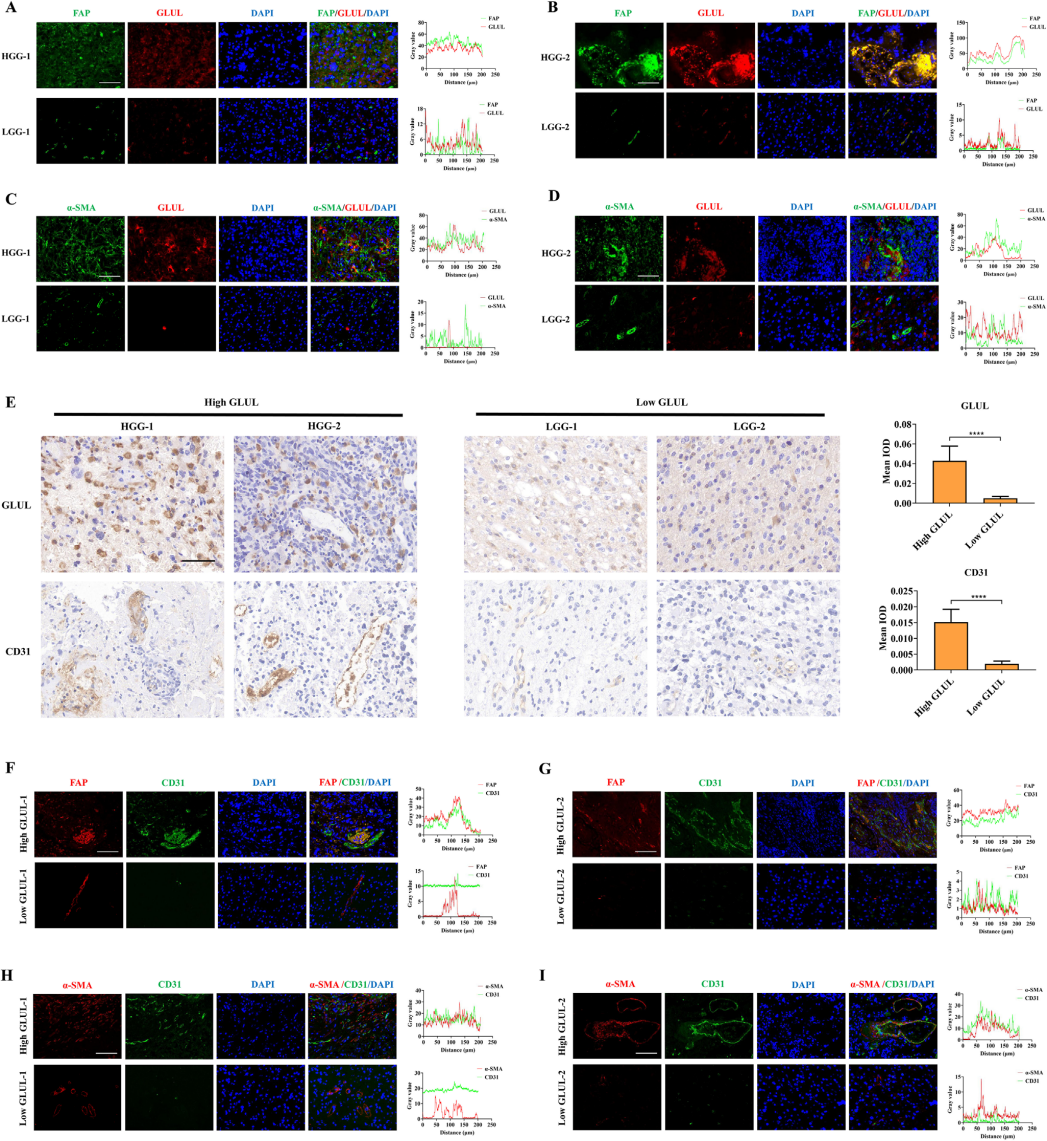
GLUL expression is upregulated and contributes to increased microvessel density in high‐grade gliomas. A,B) Representative IF images of human glioma tissues stained for FAP (green) and GLUL (red). Scale bar, 50 µm. C,D) Representative IF images of human glioma tissues stained for α‐SMA (green) and GLUL (red). Scale bar, 50 µm. E) Representative IHC images of human glioma tissues stained for GLUL and CD31 (n = 3). Scale bar, 50 µm. ^****^ *p <* 0.0001 according to the Student's *t*‐test. F,G) Representative IF images of human glioma tissues stained for FAP (red) and CD31 (green). Scale bar, 50 µm. H,I) Representative IF images of human glioma tissues stained for α‐SMA (red) and CD31 (green). Scale bar, 50 µm.

To investigate GLUL expression patterns in clinical glioma samples, immunofluorescence colocalization analysis revealed significantly greater fluorescence intensities of the FAP^+^/GLUL^+^ and α‐SMA^+^/GLUL^+^ signals in high‐grade gliomas than in their low‐grade counterparts (**Figure** **4**A–D). In high‐GLUL‐expressing glioma samples, CD31 expression was markedly increased (Figure 4E), with immunofluorescence colocalization analysis demonstrating significantly augmented coexpression signals of FAP^+^/CD31^+^ and α‐SMA^+^/CD31^+^, suggesting a potential role of GLUL in promoting CAF‐mediated glioma neovascularization (Figure 4F‐I).

### 2.3 GLUL Orchestrates CAF‐Mediated Angiogenic Competence Through the PI3K/AKT Pathway

**Figure 5:**
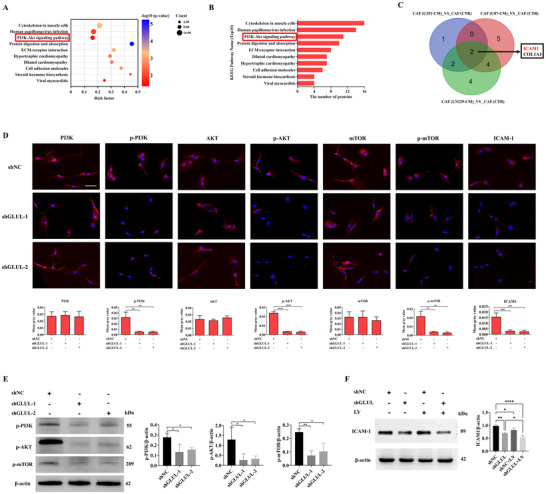
GLUL knockdown inhibits PI3K/AKT signalling in CAFs. A) KEGG pathway enrichment analysis of CAF‐related signatures. The bubble plot displays the top ten most significantly enriched pathways. The bubble size represents the number of genes, and the color intensity corresponds to the statistical significance (‐log10 (*P*‐value)) of the enrichment. B) KEGG pathway enrichment analysis revealed that the ten genes whose expression was most significantly upregulated were enriched in CAFs. C) Venn diagram showing ICAM1 as a downstream target in CAFs. D) The PI3K/AKT pathway and ICAM1 were detected via IF staining (n = 3). Scale bar, 50 µm. ^**^ *p <* 0.01, ^***^ *p <* 0.001, ^****^ *p <* 0.0001; one‐way ANOVA was used. E) Western blotting was used to detect the effect of GLUL knockdown in CAFs on the PI3K/AKT signalling pathway. F) Western blotting was used to detect the effects of GLUL knockdown or the addition of the PI3K inhibitor LY294002 on ICAM1 expression. The data are presented as the means ± SDs of triplicate wells. ^*^ *p <* 0.05, ^**^ *p <* 0.01, ^****^ *p <* 0.0001; one‐way ANOVA was used.

To mechanistically delineate GLUL downstream signaling, KEGG enrichment analysis was performed, revealing significant GLUL clustering within the PI3K/AKT signaling cascade (**Figure** **5**A,B). ICAM1 was identified as a differentially expressed adhesion molecule in CAFs (Figure 5C). Subsequent GLUL‐knockdown cells were subjected to quantitative immunofluorescence intensity profiling, which revealed marked attenuation of p‐PI3K, p‐AKT, p‐mTOR and ICAM1 expression (Figure 5D). Furthermore, western blotting confirmed that the knockdown of GLUL or the addition of the PI3K inhibitor LY294002 led to a significant downregulation of these phosphorylated proteins and reduced ICAM1 expression (Figure 5E,F), indicating the critical importance of this pathway in CAF‐mediated angiogenic activity.

**Figure 6:**
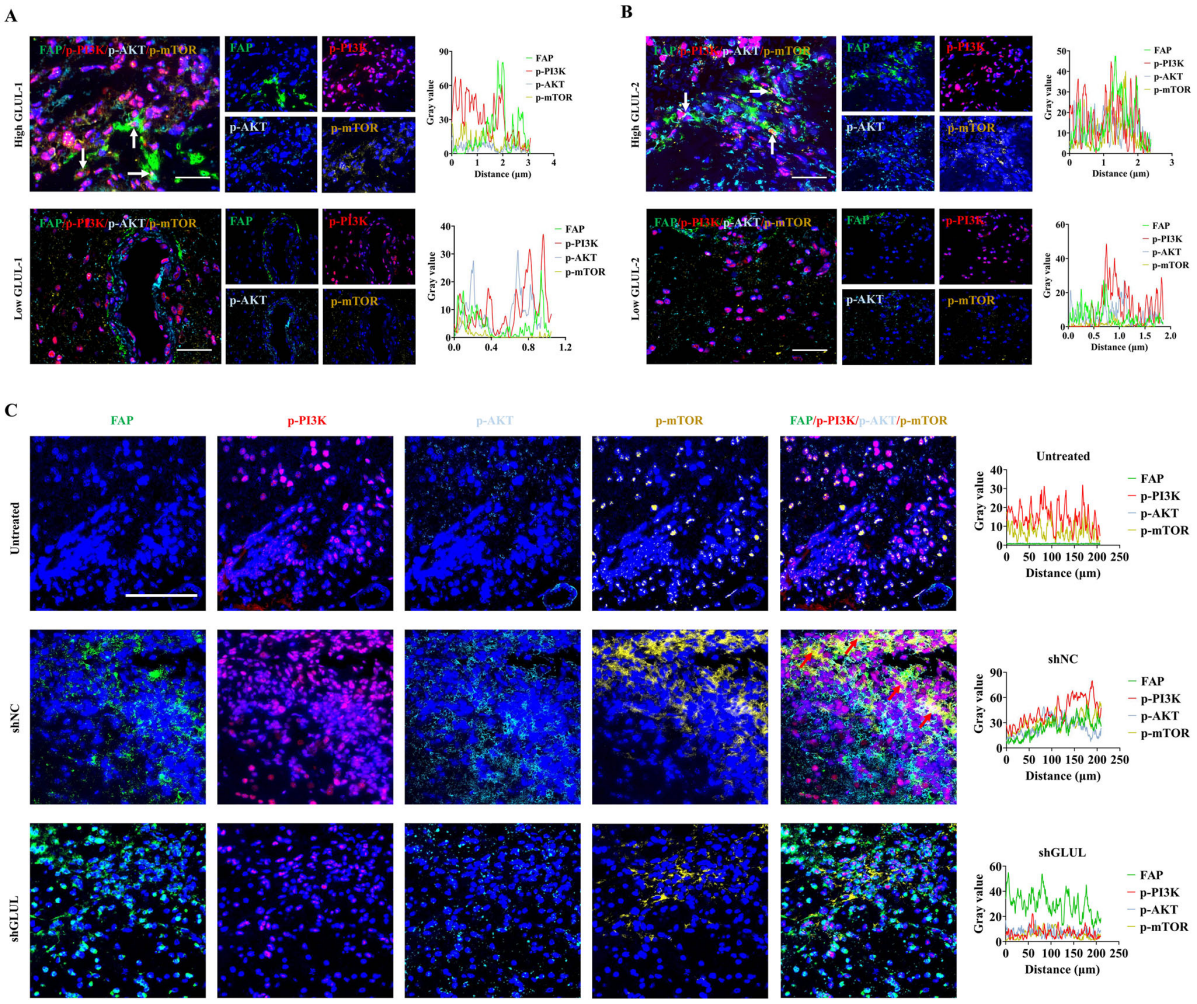
Low GLUL expression is associated with the inhibition of the PI3K/AKT pathway in clinical gliomas and orthotopic gliomas. A,B) Representative IF images of human glioma tissues stained for FAP (green), p‐PI3K (red), p‐AKT (cyan), and p‐mTOR (yellow). Scale bar, 25 µm. C) Representative IF images of orthotopic glioma tissues stained for FAP (green), p‐PI3K (red), p‐AKT (cyan), and p‐mTOR (yellow) (n = 3). Scale bar, 50 µm.

**Figure 7:**
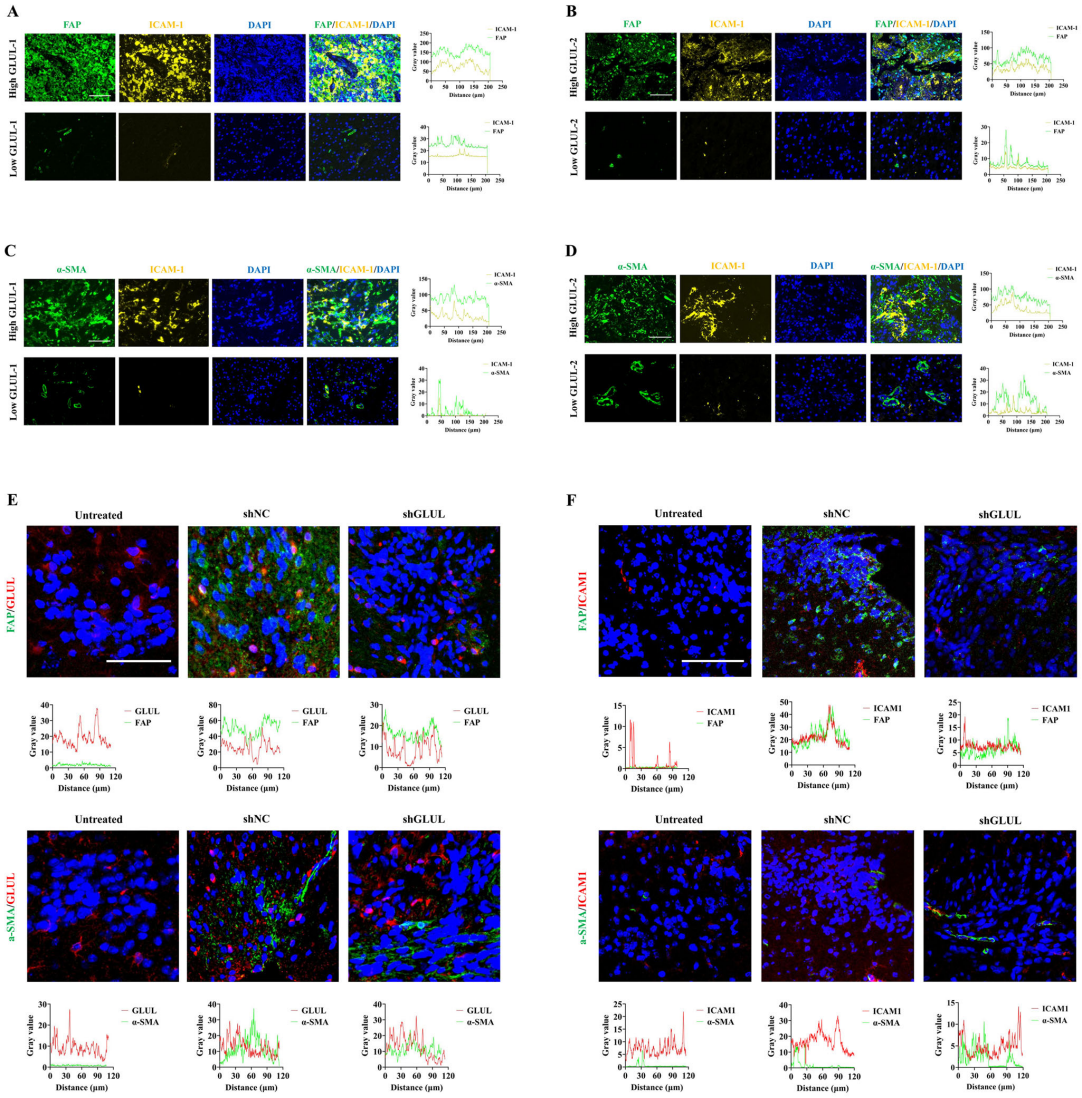
GLUL contributes to ICAM1 expression in clinical gliomas and orthotopic glioma tissues. A,B) Representative IF images of human glioma samples stained for FAP (green) and ICAM1 (yellow). Scale bar, 25 µm. C,D) Representative IF images of human glioma samples stained for α‐SMA (green) and ICAM1 (yellow). Scale bar, 25 µm. E) Representative IF images of orthotopic glioma tissues stained for FAP (green), GLUL (red), α‐SMA (green) and GLUL (red) (n = 3). Scale bar, 50 µm. F) Representative IF images of orthotopic glioma tissues stained for FAP (green), ICAM1 (red), α‐SMA (green) and ICAM1 (red) (n = 3). Scale bar, 50 µm.

In high‐GLUL‐expressing clinical samples, quantitative phosphoproteomic profiling demonstrated significantly elevated expression levels of p‐PI3K, p‐AKT, p‐mTOR, and ICAM1 compared with those in their GLUL‐low counterparts (Figure S5, Supporting Information). Multiplex immunofluorescence analysis revealed intensified coexpression signatures within the FAP^+^/p‐PI3K^+^/p‐AKT^+^/p‐mTOR^+^ molecular assemblies in high‐GLUL‐expressing glioma specimens (**Figure** **6**A,B). Similarly, GLUL knockdown in CAFs inhibited PI3K/AKT/mTOR signaling pathway activation in orthotopic glioma models (Figure 6C). Furthermore, colocalization analysis revealed that coexpression of FAP^+^/ICAM1^+^ and α‐SMA^+^/ICAM1^+^ was significantly increased in high‐GLUL‐expressing samples (**Figure** **7**A–D), and GLUL knockdown also caused downregulation of ICAM1 expression in CAFs in animal models (Figure 7E,F). Taken together, these findings suggest the potential involvement of the PI3K/AKT/ICAM signaling axis in GLUL‐driven angiogenesis in the glioma microenvironment.

### 2.4 CAFs Promote Tumor Angiogenesis in a GLUL‐Independent Manner to Fuel Tumor Growth

To mechanistically dissect GLUL‐mediated CAF‐driven tumors, we engineered in vitro coculture systems. In vitro assays revealed that the supernatant from GLUL‐silenced CAFs markedly diminished the paracrine‐driven glioblastoma proliferation capacity after 72 h (Figure S6A–C, Supporting Information). We also detected attenuated migratory potential in glioma cells exposed to CAF‐CM with GLUL knockdown (Figure S6D–F, Supporting Information). Immunofluorescence cytometric analysis revealed a diminished Ki67 proliferation index in glioma cells cultured with the supernatant derived from GLUL‐silenced CAFs (Figure S7, Supporting Information), indicating that GLUL knockdown in CAFs has a potential inhibitory effect on glioma growth.

**Figure 8:**
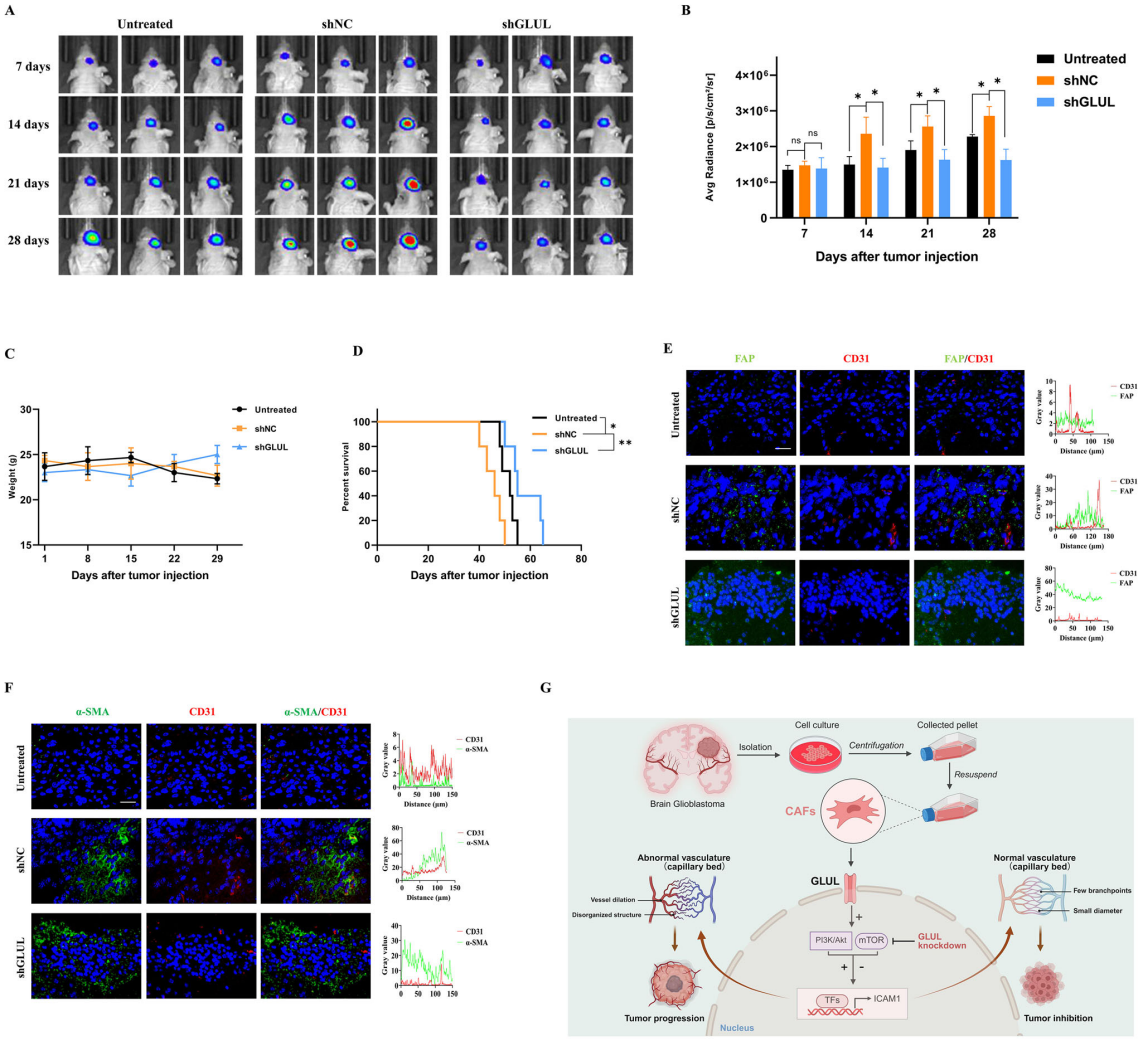
CAFs promote tumor angiogenesis through a GLUL‐independent mechanism to drive tumor growth. A) Representative images of GBM xenografts at the specified time points are displayed via bioluminescence. B) Quantification of the ROI. ^*^ *p <* 0.05 according to one‐way ANOVA. C) The body weights of the mice in the experimental groups were recorded. D) The survival of tumor‐bearing mice was monitored via Kaplan‐Meier analysis, and statistical analyses were performed with the log‐rank test. ^*^ *p <* 0.05, ^**^ *p <* 0.01. E) Representative IF images of FAP (green) and CD31 (red) expression. F) Representative IF images of α‐SMA (green) and CD31 (red) expression (n = 3). Scale bar, 25 µm. G) Schematic illustration of the proposed model. Primary CAFs were isolated and purified from fresh human GBM specimens according to established protocols. GLUL is essential for the pro‐angiogenic capacity of CAFs through its impact on the PI3K/AKT pathway. GLUL enhances the pro‐angiogenic capacity of CAFs, driving aberrant tumor vasculature that fuels tumor growth; conversely, GLUL knockdown restores vascular normalization and suppresses tumorigenesis.

To further investigate the pro‐angiogenic function and impact of GLUL‐mediated CAFs on tumor progression in vivo, we established a humanized orthotopic glioma model. In vivo imaging revealed that CAF administration contributed to glioma growth, whereas GLUL knockdown in CAFs significantly attenuated tumor progression compared with that in controls (**Figure** **8**A,B), with no significant body weight changes observed throughout the experimental period (Figure 8C). Survival analysis revealed that, compared with untreated group, control CAFs significantly reduced median survival (46 d) (52 d versus 46 d, *P =* 0.0256), whereas compared with control CAFs, GLUL‐knockdown CAFs elicited a 1.2‐fold increase in median survival duration (55 d versus 46 d, *P* = 0.0044), with all survival curves satisfying the log‐rank test assumption (Figure 8D).

Subsequently, histological staining of the tumor tissue sections was performed. H&E staining revealed morphological alterations in the tumors. Compared with the tumor‐bearing mice injected with only tumor cells, the tumor‐bearing mice injected with U251‐Luc cells mixed with GLUL‐negative control CAFs presented enhanced CD31 staining in tumor tissues; however, the tumor‐bearing mice injected with U251‐Luc cells mixed with GLUL‐silenced CAFs presented less CD31 staining in tumor tissues than that of GLUL‐negative control CAFs, and Ki67 expression was also reduced in the GLUL‐silenced CAFs (Figure S8, Supporting Information). To elucidate the spatial distribution of CAFs within the vascular niche, we performed immunofluorescence colocalization assays. These analyses revealed reduced coexpression of FAP with CD31 and α‐SMA with CD31 in tumor tissues harboring GLUL‐knockdown CAFs compared with those containing GLUL‐negative control CAFs. (Figure 8E,F).

Taken together, these findings elucidate the critical role of the GLUL/PI3K/AKT/ICAM1 axis in glioma progression by modulating CAF‐driven vascular niche remodeling, suggesting that targeted inhibition of this pathway may represent a promising therapeutic strategy for glioma (Figure 8G).

## 3 Discussion

Tumor angiogenesis is a critical driver of tumor‐promoting functions. The TME is considered to play an important role in tumor angiogenesis.^[^ ^38^, ^39^ ^]^ CAFs within the TME have been intensely investigated for their roles in promoting tumorigenesis.^[^ ^40^ ^]^ Nevertheless, the functions of CAFs in vascular remodeling within the glioma TME and the underlying molecular mechanisms remain to be elucidated. Proteomic screening identified GLUL as a differentially expressed protein in CAFs with potential vascular function. In the present study, high‐grade gliomas, particularly GBM, exhibited significant enrichment of CAFs alongside concurrent increases in GLUL expression and tumor microvascular density. Subsequent analysis demonstrated that GLUL‐knockdown CAFs display substantially impaired vasculogenic capacity with concomitant suppression of downstream PI3K‐AKT signaling. Orthotopic models confirmed attenuated tumor‐promoting functionality in GLUL‐depleted CAFs, which correlated with demonstrably extended median survival in mice. Collectively, these findings establish GLUL as both a potential biomarker for malignant progression in glioma and a promising therapeutic target in GBM.

As a major component of the tumor stroma, CAFs have the potential to remodel the immune microenvironment, promoting tumorigenic inflammation and immunosuppression.^[^ ^10^, ^41^, ^42^ ^]^ In breast cancer, CAFs additionally exhibit vasculogenic and vessel permeability‐inducing capabilities, thereby driving tumor growth and metastasis.^[^ ^43^ ^]^ This finding is consistent with our prior work demonstrating CAF‐mediated remodeling of the glioma immune microenvironment and the critical importance of CAF‐induced immunosuppression in glioma.^[^ ^37^ ^]^ Although the protumorigenic and ECM‐remodeling functions of CAFs have been extensively studied and recognized,^[^ ^44^, ^45^ ^]^ research concerning their involvement in glioma vascularization remains relatively scarce. We have shown that CAFs within the glioma microenvironment establish stable vascular‐like structures and induce endothelial cells to participate in vasculogenic mimicry. However, the molecular mechanisms by which CAFs contribute to glioma angiogenesis remain poorly characterized. Our proteomics analysis revealed differentially expressed proteins in CAFs and identified GLUL as a potential regulator of vasculature development. GLUL has been predominantly implicated in tumor metabolic reprogramming,^[^ ^32^, ^46^ ^]^ however, its role in the tumor vasculature has rarely been explored. Herein, we report that GLUL is crucial for CAF‐mediated angiogenesis in glioma.

In addition, examining how CAF metabolism serves as a critical bridge to angiogenesis via the lactate shuttle, glutamine recycling, and noncanonical pathways would offer a more unified and mechanistic perspective. Previous studies have conclusively shown that the angiogenic function of GLUL in endothelial cells is intrinsically linked to its enzymatic activity, which supports endothelial cell proliferation and survival by maintaining intracellular glutamine levels.^[^ ^36^ ^]^ Our results reveal a previously undescribed mechanism. We showed for the first time that CM from GLUL‐expressing CAFs was sufficient to induce angiogenesis, suggesting that a secreted factor is responsible for this process. Furthermore, GLUL attenuates CAF function via PI3K/AKT inhibition, and the expression level of GLUL in CAFs determines the malignant potential of clinical glioma samples, suggesting that CAF‐targeted glioma treatment is possible. Functional validation demonstrated that GLUL knockdown in CAFs significantly impaired their angiogenic capacity and suppressed orthotopic glioma growth in mice. Moreover, clinical glioma specimens revealed that elevated GLUL expression was correlated with increased microvascular density.

Critically, cohigh expression of FAP/GLUL or α‐SMA/GLUL marked tumors with advanced malignancy, indicating that GLUL contributes to malignant progression. Previous studies established the PI3K/AKT signaling pathway as a critical player in tumor angiogenesis.^[^ ^47^, ^48^ ^]^ Furthermore, KEGG pathway analysis pinpointed GLUL enrichment within the PI3K/AKT pathway, whereas ICAM1, as an adhesion molecule, contributes to vascular homeostasis maintenance. Strikingly, we found that GLUL knockdown in CAFs suppressed PI3K/AKT/mTOR signaling, as evidenced by decreased expression of p‐PI3K, p‐AKT, p‐mTOR, and ICAM1 in CAFs. Moreover, the PI3K inhibitor LY294002 also reduced ICAM1 expression in CAFs, which confirmed that the PI3K/AKT pathway was involved in the GLUL‐mediated angiogenesis of CAFs. Subsequent investigations in animal models revealed that GLUL knockdown also reduced the expression of corresponding phosphorylated proteins within the PI3K/AKT/mTOR pathway and of ICAM1. Moreover, phosphoprotein expression was significantly stronger in tumor samples with high GLUL levels than in those with low expression, suggesting the potential involvement of this pathway in the GLUL‐mediated pro‐angiogenic and tumor‐promoting functions of CAFs. Given that GBM exhibits both aberrant vascularization and a profoundly immunosuppressive microenvironment,^[^ ^49^, ^50^ ^]^ the potential role of CAFs in glioma immune remodeling warrants further investigation. CAF‐induced abnormal angiogenesis may lead to dysfunctional blood vessels, impairing the infiltration and efficacy of cytotoxic T lymphocytes. Furthermore, hypoxic signaling triggered by this aberrant vasculature may amplify the expression of immunosuppressive factors by CAFs, thereby reinforcing immune evasion. Therefore, elucidating the mechanisms underlying CAF‐mediated vascular‐immune crosstalk will identify novel therapeutic targets to normalize the GBM microenvironment and overcome treatment resistance.

This study has several limitations. For example, the specific mechanism by which CAF‐related proteins regulate glioma vascularization needs to be further explored. Furthermore, the expression of GLUL in CAFs suggests its malignant potential in human glioma tissues, and the number of clinical samples was relatively small; thus, subsequent work must include the validation of expanded sample cohorts. Despite the novel contributions of our studies to CAF‐mediated glioma angiogenesis, the heterogeneity of CAFs and the lack of specific markers present potential pitfalls for research in this field. Future studies will focus on delineating the heterogeneity of glioma‐derived CAFs through single‐cell multiomics sequencing. Defining the specific transcriptional subtypes that drive vascular mimicry and other protumor functions will be critical for developing targeted stromal therapies. More novel approaches for the detection of CAFs need to be applied, aiming to develop standardized and individualized therapeutic strategies for glioma. To investigate the GLUL‐mediated tumor‐promoting functions of CAFs, proteomic landscapes revealed differential protein expression in glioma microenvironment‐reprogrammed CAFs, underscoring GLUL as a critical element in glioma progression. Taken together, our findings revealed the pro‐angiogenic capacity of GLUL‐driven CAFs and their potential clinical significance in defining novel therapeutic targets for glioma.

## 4 Conclusion

This study revealed that GLUL critically shapes CAF functionality to promote angiogenesis and drive a protumorigenic phenotype. These findings point to a critical role for GLUL‐driven activation of PI3K/AKT signaling in regulating CAF‐mediated formation of the vascular niche in glioma. Furthermore, our investigation revealed that targeting GLUL in CAFs is an innovative stromal‐centric therapeutic strategy for GBM treatment.

## 5 Experimental Section

This study complies with all relevant ethical regulations. All animal experiments were approved by the Beijing Neurosurgical Institute. All human donors/patients provided informed consent, and the study was approved by Beijing Chaoyang Hospital, Capital Medical University.

### Cell Culture

Glioma‐derived primary CAFs were obtained following standardized methodological procedures.^[^ ^37^ ^]^ LN229, U87, and U251 glioma cells were cultured in DMEM (Gibco, USA) supplemented with 10% fetal bovine serum (FBS; Invitrogen, China) and 100 U mL^−1^ penicillin/streptomycin (Gibco, USA). The above cells were incubated at 37 °C with 5% CO_2_.

### Preparation of CM

Glioma cells and CAFs were seeded in plates containing 10% FBS (Invitrogen, China) and 100 U mL^−1^ penicillin/streptomycin (Gibco, USA) and then cultured for 72 h. The cell supernatants were centrifuged at 2000 rpm for 10 min to obtain CM from all the cells.

### Proteomic Sample Preparation

CAFs were incubated for 72 h with different types of glioma CM (U87‐CM, U251‐CM, and LN229‐CM), and untreated cells were used as controls. All the cells were subsequently collected for proteomic analysis according to the manufacturer's recommendation in Annoroad Gene Technology. Briefly, protein extraction was performed via SDT lysis buffer (4% SDS, 100 mm Tris‐HCl, pH 7.6), and the protein concentration was quantified with a BCA protein assay kit (Bio‐Rad, USA). For digestion, protein samples were reduced with 10 mm DTT, alkylated with 20 mM iodoacetamide in the dark, and digested with trypsin (enzyme‐to‐protein ratio of 1:50) overnight at 37 °C via a filter‐aided sample preparation (FASP) protocol. The resulting peptides were desalted via C18 StageTips, concentrated via vacuum centrifugation, and reconstituted in 0.1% formic acid.

### LC‐MS/MS Analysis

LC‐MS/MS analysis was conducted via a timsTOF Pro mass spectrometer (Bruker) coupled with a NanoElute nano‐UHPLC system (Bruker). Peptides were loaded onto a C18 reversed‐phase analytical column (Thermo Scientific Easy Column, 25 cm × 75 µm i.d., 1.9 µm particle size) with an initial mobile phase consisting of 95% buffer A (0.1% formic acid in water). Separation was achieved via a linear gradient of buffer B (99.9% acetonitrile, 0.1% formic acid) at a constant flow rate of 300 nL min^−1^. The mass spectrometer was operated in positive ion mode with an electrospray voltage of 1.5 kV. Both precursor and fragment ions were detected across a mass range of m/z 100–1700. The instrument was operated in parallel accumulation serial fragmentation (PASEF) mode, with ion mobility coefficients (1/K0) ranging from 0.6 to 1.6 Vs cm^2^.

### Identification and Quantification Of Proteins

Data acquisition included one full MS scan followed by ten PASEF MS/MS scans. Active exclusion was enabled with a 24‐s release interval. The raw MS data for each sample were combined and searched via MaxQuant 1.6.14 software for identification and quantitative analysis. The criterion for identifying significantly differentially expressed proteins was set as a fold change (FC) > 2.0 (upregulated > 2.0 or downregulated < 0.5) with a *P*‐value < 0.05 (from a *t*‐test or other statistical tests). On the basis of these criteria, the numbers of up‐ and downregulated proteins in the comparative groups were determined.

### Functional Enrichment Analysis

The protein sequences of the differentially expressed proteins were aligned against the InterPro and NCBI nonredundant databases via BLAST+ and InterProScan to assign gene ontology (GO) terms via Blast2GO. GO term distributions were visualized via custom R scripts. For pathway analysis, proteins were searched via the Kyoto Encyclopedia of Genes and Genomes (KEGG) database (https://www.kegg.jp/) to assign KEGG orthology identifiers and map them to biological pathways. Functional enrichment analysis for GO terms and KEGG pathways was performed via one‐sided Fisher's exact test, with the full set of quantified proteins as the background. The resulting *P*‐values were adjusted for multiple comparisons via the Benjamini‐Hochberg procedure. Terms with an adjusted *P*‐value < 0.05 were considered statistically significant.

### RNA Interference, Plasmids, and Reagents

GLUL‐knockdown cells were produced via lentivirus‐mediated transduction via synthetic short hairpin RNA (shRNA) oligonucleotides (GeneChem, Shanghai, China) according to the manufacturer's protocols. The sequences of the shRNAs used are listed in Table S1 (Supporting Information).

### Flow Cytometry

CAFs were collected for flow cytometry analysis. Briefly, all the cells were fixed with 4% PFA and permeabilized with Flow Cytometry Perm Buffer (BD Biosciences, USA). Then, the cell suspensions were stained with antibodies against human FAP (1:20, R&D, USA), a‐SMA (1:100, Abcam, USA), and GLUL (1:500, Proteintech, USA) and incubated in the dark at 4 °C for 30 min. Then, the cells were centrifuged and resuspended in PBS. All the data were acquired via a BD Accuri C6 Plus (BD Biosciences) and analyzed via FlowJo software (Tree Star, Ashland, OR, USA).

### Cell Viability

Glioma cells were subjected to different conditions for 24, 48, or 72 h. CCK‐8 solution was added to each well, and the samples were incubated for 2 h at 37 °C. The absorbance of each well at 450 nm was subsequently measured with a microplate reader (Biotek, MQX200, Winooski, VT, USA) to calculate cell viability.

### Transwell Migration Assay

The cells were suspended in FBS‐free DMEM and added to the upper chamber, and the bottom chamber was filled with different media. After co‐incubation for 24 h, the cells were fixed and stained. The migrated cells were viewed with a laser‐scanning confocal microscope (LSCM; Leica, Germany) and processed via ImageJ software.

### Tube Formation Assay

The growth factor‐reduced Matrigel basement membrane matrix (Corning, USA) was slowly thawed on ice, and 50 µL of Matrigel was added to each well of a 96‐well plate and incubated at 37 °C for 40 min. CAFs and HUVECs were trypsinized and seeded on a Matrigel matrix at a density of 2 × 10^4^ cells per well. Then, the cells were stained with 5 µm calcein AM (Tocris, USA). Additionally, DIL‐labeled CAFs and DIO‐labeled HUVECs were seeded in additional wells at a 1:1 ratio. After incubation at 37 °C for 8 h, images were captured with an Olympus microscope and analyzed via ImageJ software.

### Immunofluorescence

CAFs grown on glass coverslips were fixed with 4% paraformaldehyde for 15 minutes, permeabilized with 1% Triton X‐100 for 15 minutes, and blocked with 2.5% bovine serum albumin (BSA)‐PBST solution for 1 h at room temperature. Then, the cells were incubated with FAP (1:100, Abcam, ab314456), α‐SMA (1:100, Abcam, ab124964), GLUL (1:800, Abcam, ab73593), PI3K (1:200, Proteintech, China), p‐PI3K (1:200, SAB, Catalog No: #11508), AKT (1:400, Proteintech, Catalog No: 60203‐2‐Ig), p‐AKT (1:200, Proteintech, Catalog No: 66444‐1‐Ig), mTOR (1:600, Proteintech, Catalog No: 66888‐1‐Ig), p‐mTOR (1:200, Proteintech, Catalog No: 67778‐1‐Ig), and ICAM1 (1:100, Abcam, ab109361) at 4 °C overnight. Then, the cells were incubated with fluorescence‐labeled secondary antibodies for 1 h, and the nuclei were stained with DAPI for 10 min. All images were acquired under a fluorescence microscope.

### RT‐qPCR

Total RNA was isolated via a Macherey‐Nagel kit, and complementary DNA was synthesized via a high‐capacity RNA‐to‐cDNA kit according to the manufacturer's instructions. RT‐qPCR was performed with Power SYBR Green PCR master mix. For data analysis, the QuantStudio 6 Flex Real‐Time PCR System (Applied Biosystems) was used. Relative gene expression levels were defined via the DDCt method, and normalization was performed to 18S rRNA. All primers used in this study are described in Table S2 (Supporting Information).

### Immunohistochemistry

The tumor tissues were collected and fixed with 4% PFA, embedded in paraffin, sectioned, and stained with hematoxylin and eosin (H&E). For immunohistochemical staining, paraffin‐embedded sections were subjected to antigen heat retrieval with citric acid (pH 6.0). The sections were incubated with primary antibodies against FAP (1:1000, Abcam, ab314456), a‐SMA (1:1500, Abcam, ab124964), GLUL (1:1000, Abcam, ab73593), CD31 (1:300, Abcam, ab76533), p‐PI3K (1:200, SAB, Catalog No: #11508), p‐AKT (1:400, Proteintech, Catalog No: 66444‐1‐Ig), p‐mTOR (1:1000, Proteintech, Catalog No: 67778‐1‐Ig), and ICAM1 (1:200, Abcam, ab109361) overnight at 4 °C and then with secondary antibodies for 1 h, followed by incubation with HRP (1:200) for 50 min. A DAB substrate kit was used as a chromogen. The tissue sections were counterstained with hematoxylin, dehydrated, and mounted.

### Western Blot Analysis

CAFs were pretreated with lentivirus‐mediated GLUL knockdown or the PI3K inhibitor LY294002 (MCE, HY‐10108) at 0.3 µm. All the cells were collected in ice‐cold PBS, and proteins were extracted via 1X RIPA buffer supplemented with protease (Sigma, B8640) and phosphatase inhibitors (Sigma, P5726 and P0044). Proteins were separated by 4–15% SDS‐PAGE, transferred to polyvinylidene fluoride (PVDF) membranes, and blocked with 5% nonfat milk or BSA in 1X PBS with Tween‐20 (0.1%). Primary antibodies against GLUL (1:1000, Abcam, ab73593), p‐PI3K (1:800, SAB, Catalogue No: #11508), p‐AKT (1:5000, Proteintech, Catalogue No:66444‐1‐Ig), p‐mTOR (1:5000, Proteintech, Catalogue No: 67778‐1‐Ig), and ICAM1 (1:4000, Abcam, ab109361) were used. After incubation with secondary antibodies for 60 min, the blots were washed, incubated with SuperSignal WestFemto Maximum Sensitivity Substrate (Thermo Fisher Scientific), and analyzed via a ChemiDoc^TM^ Touch Imaging System (Bio‐Rad, Hercules, CA, USA). For the densitometric analysis of the western blots, ImageJ software (version 1.42, National Institutes of Health, USA) was used.

### Tissue fluorescence staining

The tissue sections were first blocked with 3% hydrogen peroxide solution to block endogenous peroxidase activity and then blocked with 3% BSA. Primary antibodies against FAP (1:100, Abcam, ab314456), a‐SMA (1:400, Abcam, ab124964), GLUL (1:1000, Abcam, ab73593), CD31 (1:500, Abcam, ab76533), p‐PI3K (1:200, SAB, Catalog No: #11508), p‐AKT (1:200, Proteintech, Catalog No:66444‐1‐Ig), p‐mTOR (1:200, Proteintech, Catalog No: 67778‐1‐Ig), and ICAM1 (1:100, Abcam, ab109361) were used. The samples were incubated overnight at 4 °C. After three washes with TBS‐T, the samples were incubated with an HRP‐conjugated secondary antibody at room temperature for 50 min. Fluorescence scanning was performed after DAPI staining.

### Establishment of an Orthotopic Glioma Model

All animal procedures were approved by the Experimental Animal Ethics Committee of the Beijing Neurosurgical Institute (Approval No. BNI202307009), and all experiments were conducted in accordance with institutional and national guidelines.

Six‐week‐old male BALB/c nude mice were purchased from GemPharmatech Co., Ltd. (Jiangsu, China) and housed under specific pathogen‐free (SPF) conditions with free access to food and water. The mice were randomly assigned to three groups (n = 6 per group): untreated, shNC, and shGLUL‐1. Following anesthesia with isoflurane, intracranial injections were performed in the right striatum at coordinates 2 mm lateral and 1 mm anterior to the brainstem at a depth of 3 mm from the cortical surface.

In the untreated group, 2 × 10⁵ U251‐luciferase (U251‐Luc) cells were injected. In the shNC group, 2 × 10⁵ U251‐Luc cells were coinjected with 5 × 10⁴ control fibroblasts. In the shGLUL‐1 group, 2 × 10⁵ U251‐Luc cells were coinjected with 5 × 10⁴ GLUL‐1‐knockdown fibroblasts. No additional treatment was administered after cell implantation. Each mouse was treated with 100 µL of 15 mg mL^−1^ D‐luciferin (PerkinElmer, Waltham, MA, USA) intraperitoneally (i.p.) in PBS. Tumor progression was monitored via an IVIS Spectrum imaging system (PerkinElmer) on d 7, 14, 21, and 28 post‐injection. Images were captured, and the region of interest (ROI) was acquired. Body weight and survival status were recorded weekly. At the endpoint, brain tissues from one representative mouse per group were collected for hematoxylin and eosin (H&E) staining, immunohistochemistry (IHC), and immunofluorescence analysis.

### Statistical Analysis

Statistical analysis was performed via GraphPad Prism 10.0 software. The data were presented as the mean±SD. Each in vitro experiment was independently repeated at least three times. Differences between groups were compared via Student's *t*‐test or one‐way ANOVA. The data distribution was assumed to be normal, but this was not formally tested. Randomization of animal studies was used in the data analysis. Kaplan–Meier analysis was conducted to assess the overall survival rate. Comparisons between curves were performed via the log‐rank test. Data collection and analysis were not performed in a blinded manner to the conditions of the experiments. Statistical significance was set at *p <* 0.05. The notations ^*^ *p <* 0.05, ^**^ *p <* 0.01, ^***^ *p <* 0.001, ^****^ *p <* 0.0001.

## Conflict of Interest

The authors declare no conflict of interest.

## Author Contributions

Q.Z. and Y.L. contributed equally to this work. Q.Z. conceived and designed the study and wrote the paper. Y.W. and F.L. supervised the study. Q.Z. performed most of the experiments, including cell biology, biochemical, and flow cytometry analyses. Y.L. conducted the animal studies. Z.Z. was involved in the cell culture. Q.Z. and F.L. provided funding support and revised the paper. All the authors read and edited the manuscript.

## Supporting information



Supporting Information

## Data Availability

The data that support the findings of this study are available from the corresponding author upon reasonable request.
