# Supplementary material for: GLUL Confers Perivascular Cancer‐Associated Fibroblasts With Pro‐Angiogenic Capacity to Promote Glioma Progression
Source: Adv Sci (Weinh). 2025 Dec 8;13(8):e13184. doi: 10.1002/advs.202513184 (PMC12884781; doi:10.1002/advs.202513184)
Supplement: Supplementary file 1 — Supporting Information [file ADVS-13-e13184-s001.docx]

**Supplementary figures**


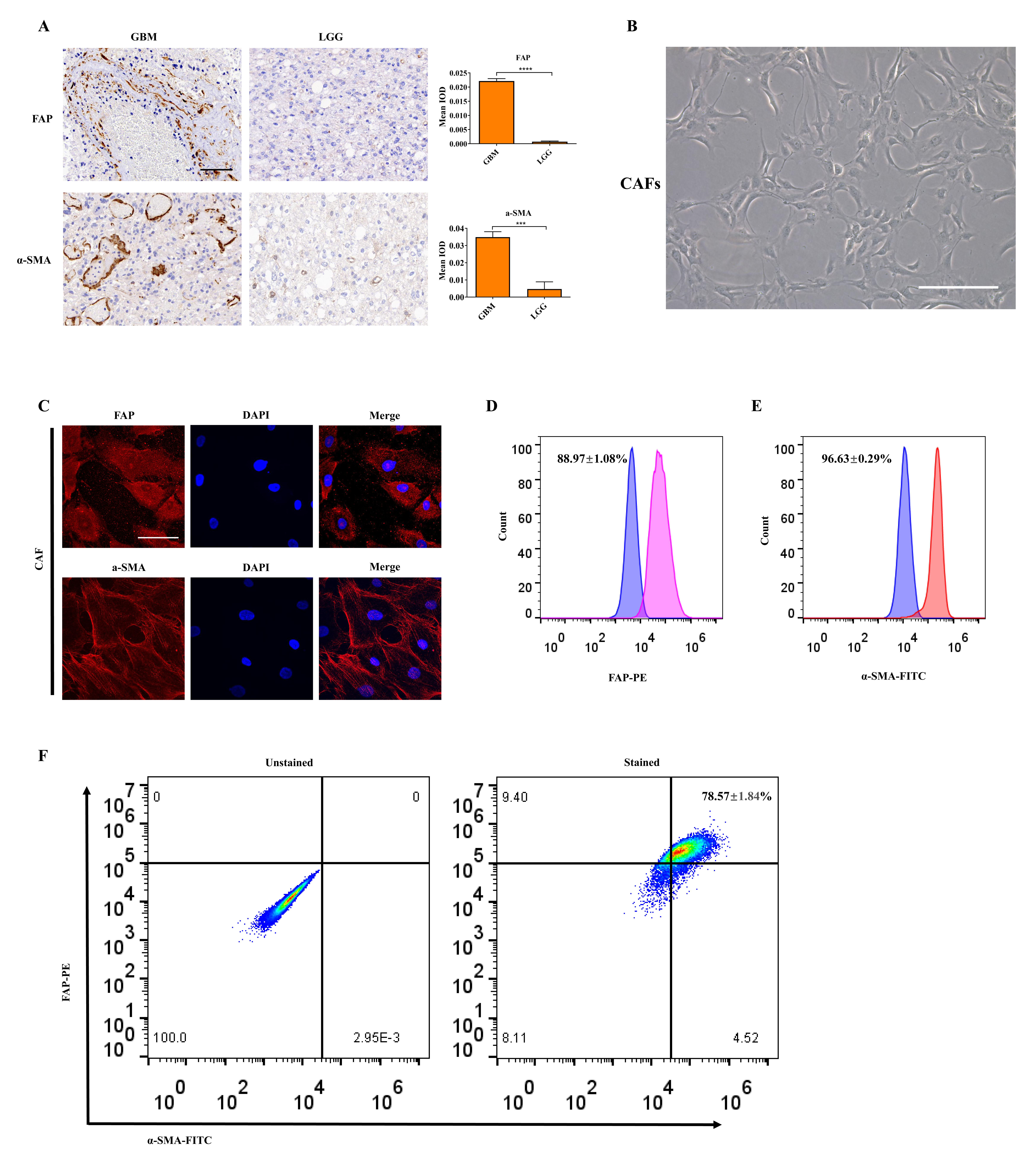


**Figure S1.** **Identification of primary CAFs from human glioma specimens.** A. Representative IHC images from human glioma tissues stained for FAP and a-SMA (n=3). Scale bar, 50 μm. ****P* < 0.001, *****P* < 0.0001 using one-way ANOVA. B. Morphological images of the in vitro cultured CAFs. Scale bar, 500 μm. C. Representative IF images from primary cells stained for FAP and a-SMA (n=3). Scale bar, 200 μm. D-F. FAP and a-SMA levels were detected by flow cytometry in the indicated cells (n=3).


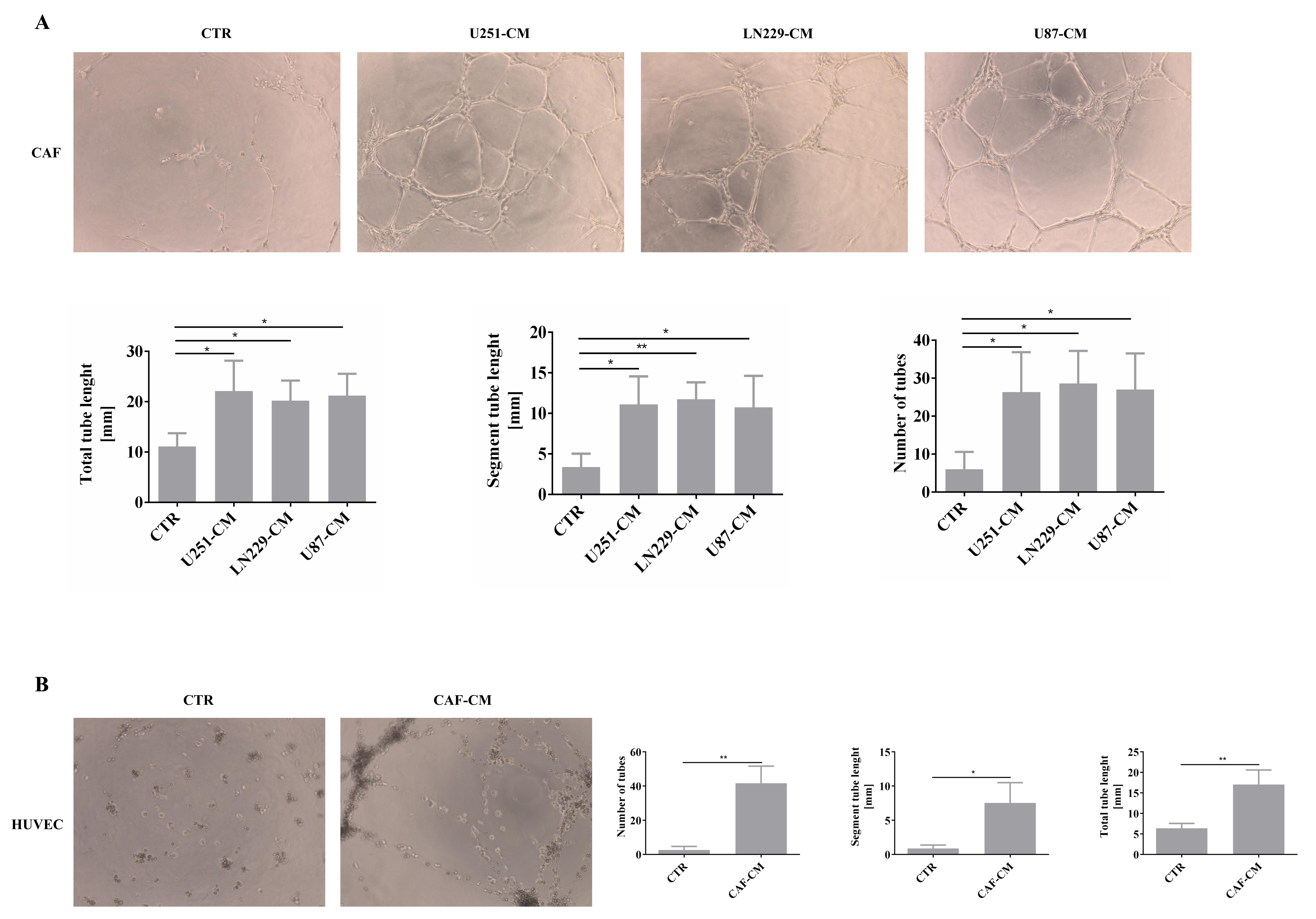


**Figure S2. Angiogenic capacity of CAFs for 24 h.** A. Tube formation capacity of CAFs cultured with glioma CM (n=3). Scale bar, 500 μm. **P* < 0.05, ***P* < 0.01 using one-way ANOVA. B. Tube formation capacity of HUVECs cultured with CAF-CM (n=3). Scale bar, 500 μm. **P* < 0.05, ***P* < 0.01 using one-way ANOVA.


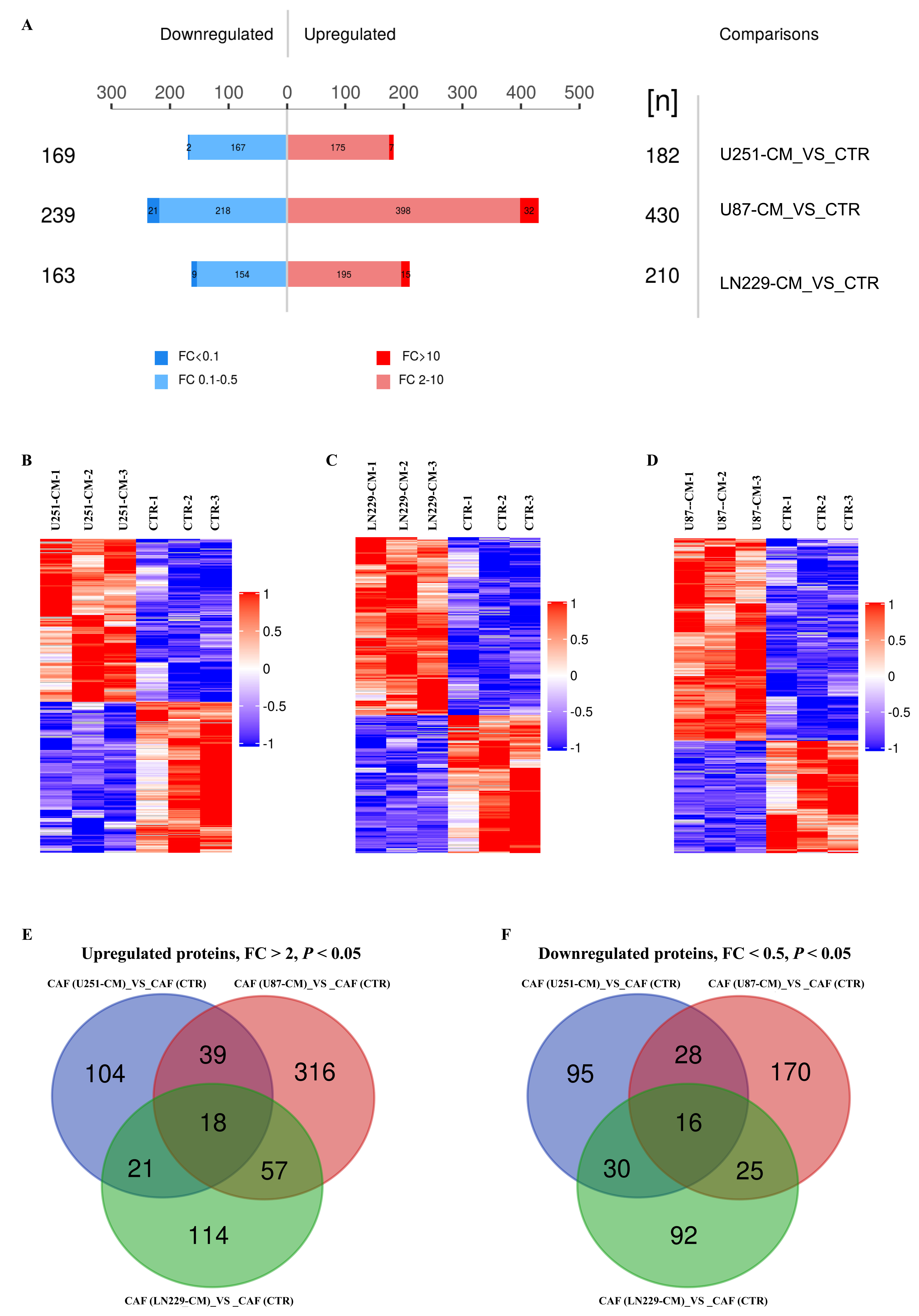


**Figure S3. Proteomic analysis displays statistically differential proteins in CAFs.** A. The number of Upregulated and Downregulated proteins. B-D. The significantly altered proteins were identified in primary CAFs. E. Venn diagram of upregulated proteins. FC > 2, *P* < 0.05. F. Venn diagram of downregulated proteins. FC < 0.5, *P* < 0.05.


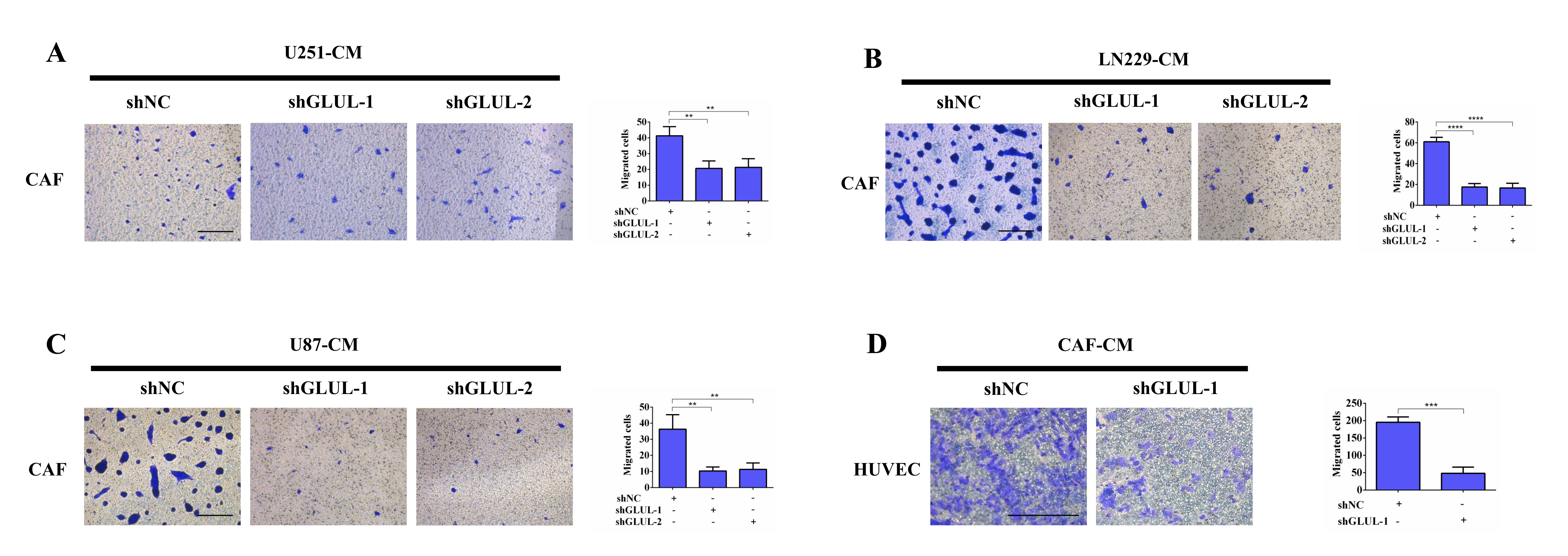


**Figure S4. Migration of CAFs and HUVECs in different conditions** for 24 h**.** A. Representative images from the migration of CAFs under U251-CM (n=3). Scale bar, 2000 μm. ***P* < 0.01 using one-way ANOVA. B. LN229-CM (n=3). Scale bar, 2000 μm. *****P* < 0.0001 using one-way ANOVA. C. U87-CM (n=3). Scale bar, 2000 μm. ***P* < 0.01 using one-way ANOVA. D. Representative images from the migration of HUVECs under CAF-CM (n=3). Scale bar, 2000 μm. ****P* < 0.001 using one-way ANOVA.


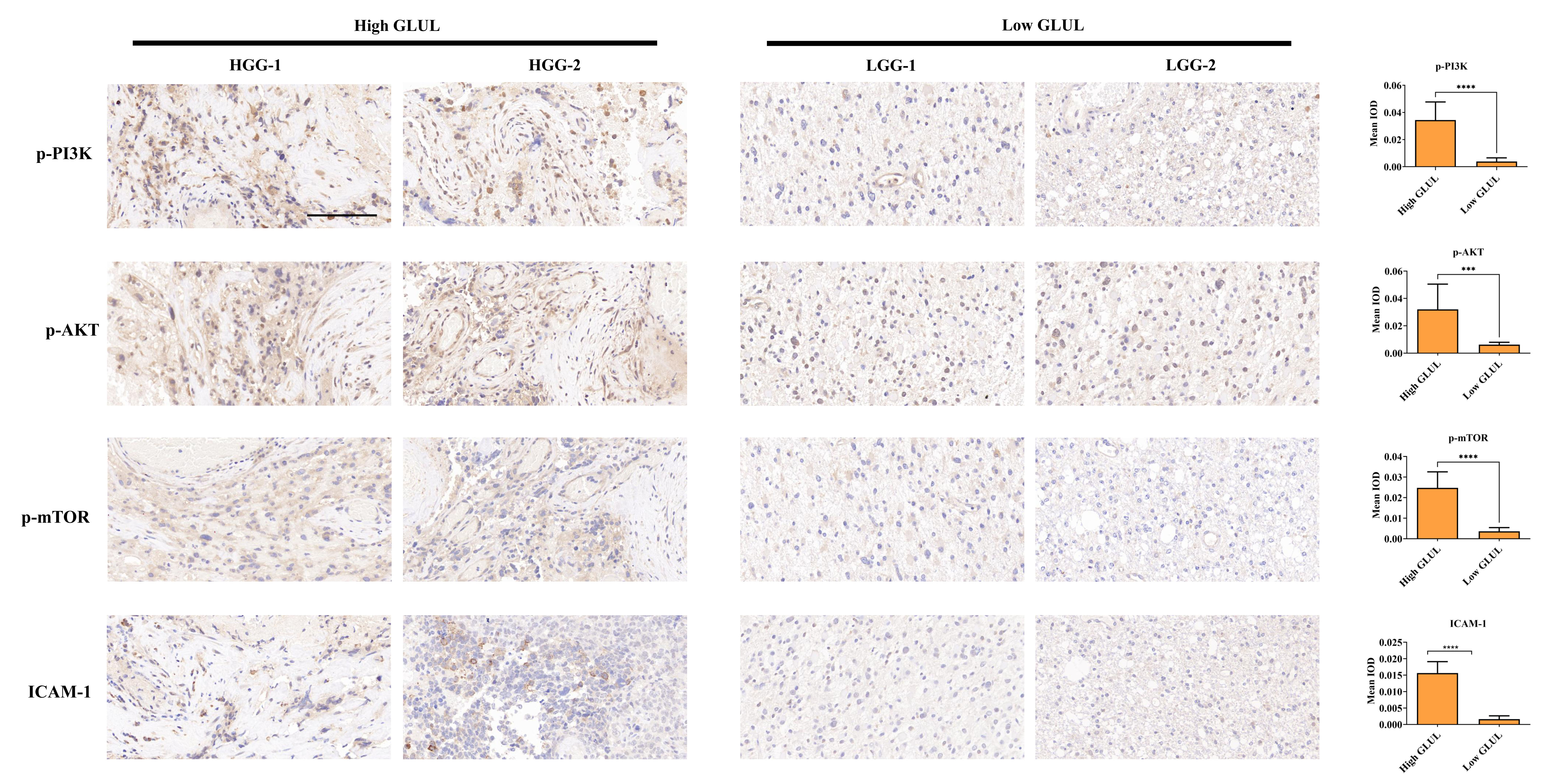


**Figure S5. The expression of p-PI3K, p-AKT, p-mTOR and ICAM1 in clinical gliomas.** Representative IHC images are shown （n=3）. Scale bar, 50 μm. ****P* < 0.001, *****P* < 0.0001 using Student’s *t* test.


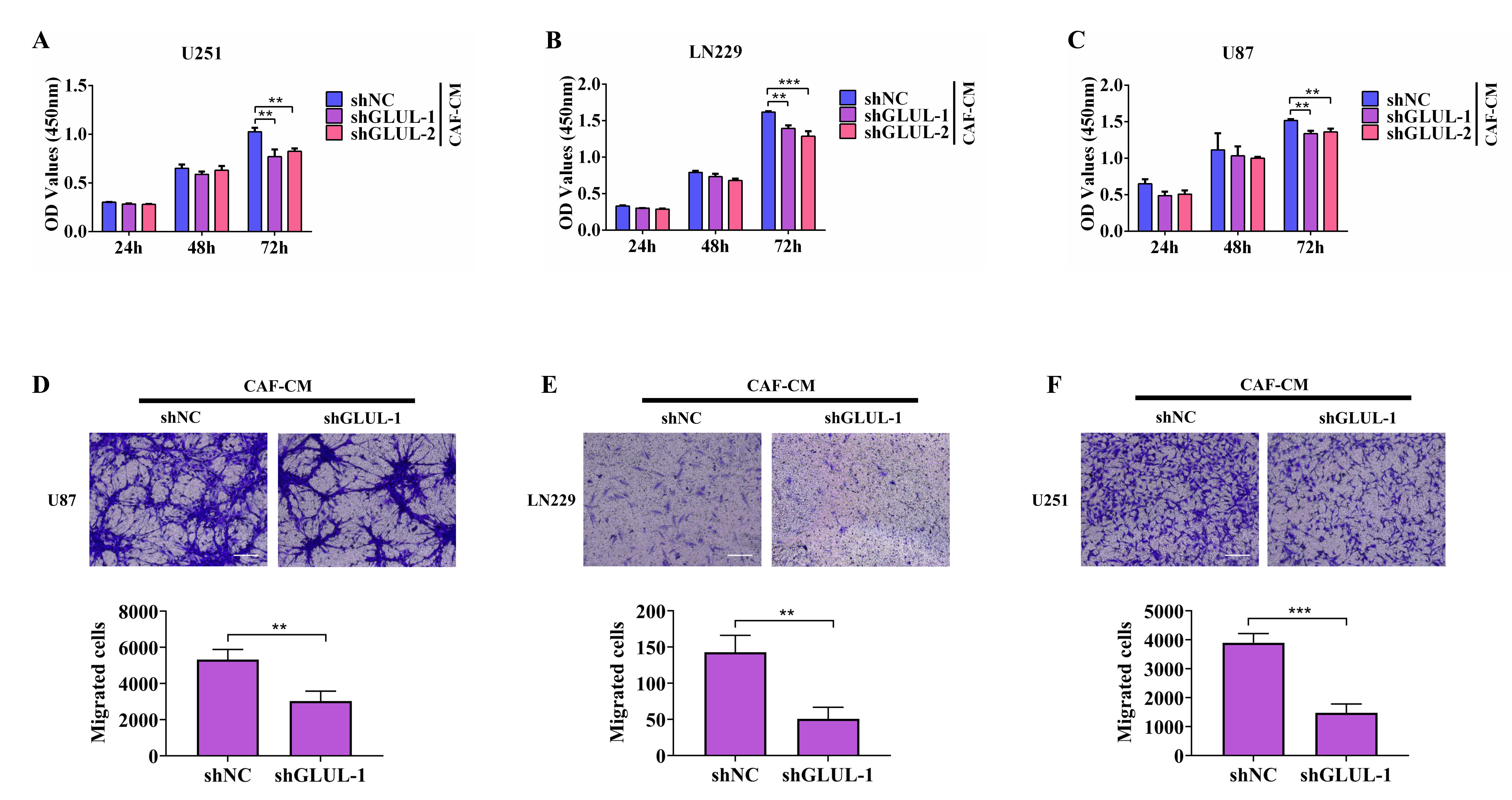


**Figure S6. GLUL-knockdown CAFs reduce the proliferation and migration of glioma cells.** A. CCK8-assay evaluated the effects of conditioned media from CAF with GLUL knockdown on U251 proliferation (n=5). ***P* < 0.01 using one-way ANOVA. B. LN229 (n=5). ***P* < 0.01, ****P* < 0.001 using one-way ANOVA. C. U87 (n=5). ***P* < 0.01 using one-way ANOVA. D-F. Transwell assay evaluated the effects of conditioned media from CAF with GLUL knockdown on U87, LN229, and U251 migration for 24 h (n=3). Scale bar, 2000 μm. ***P* < 0.01, ****P* < 0.001 using Student’s *t* test.


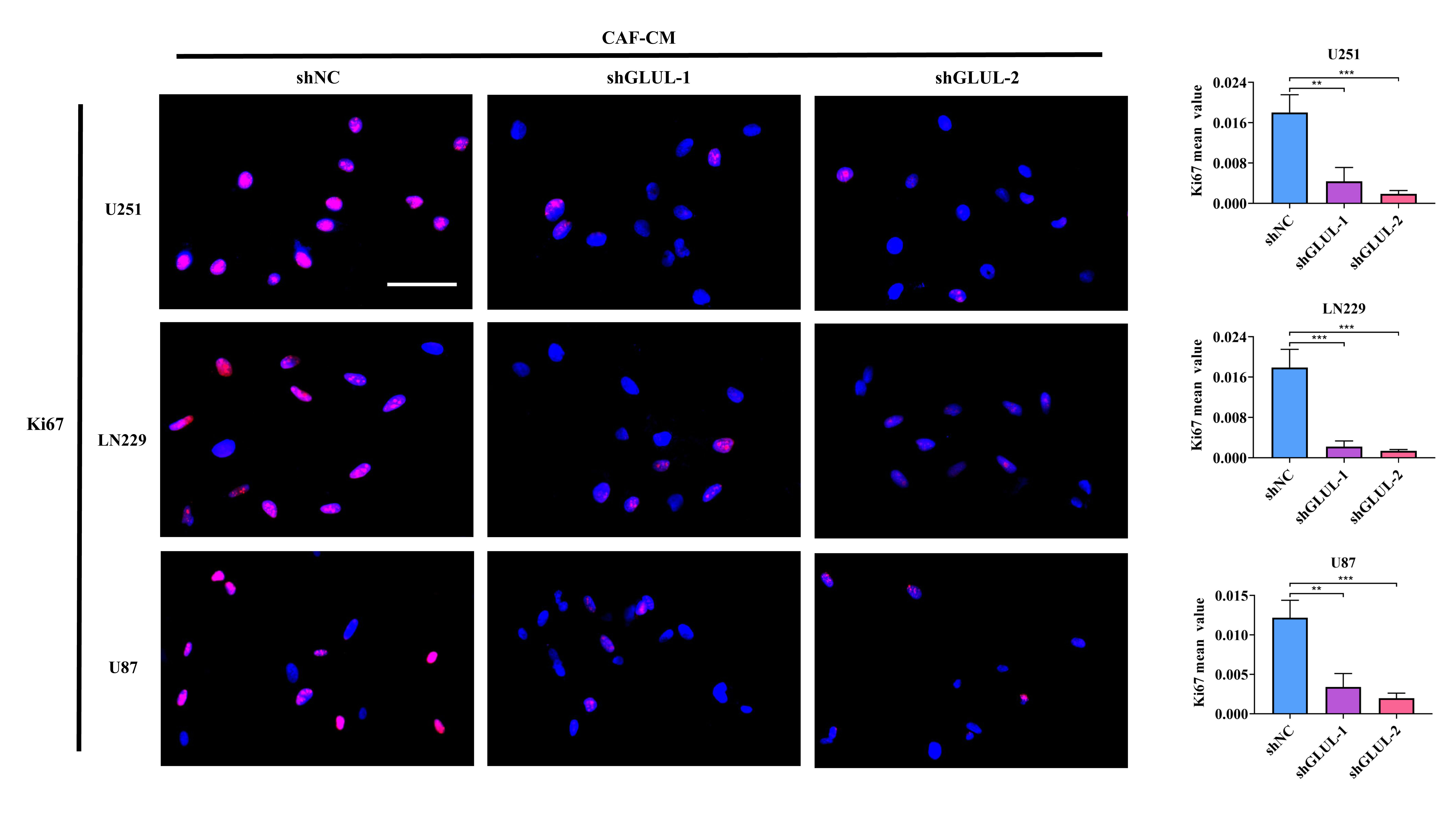


**Figure S7. GLUL-knockdown CAFs attenuate Ki67 expression in glioma cells.** Representative IF images from Ki67 expression of U251, LN229 and U87 (n=3). Scale bar, 50 μm. **P < 0.01, ***P < 0.001 using one-way ANOVA.


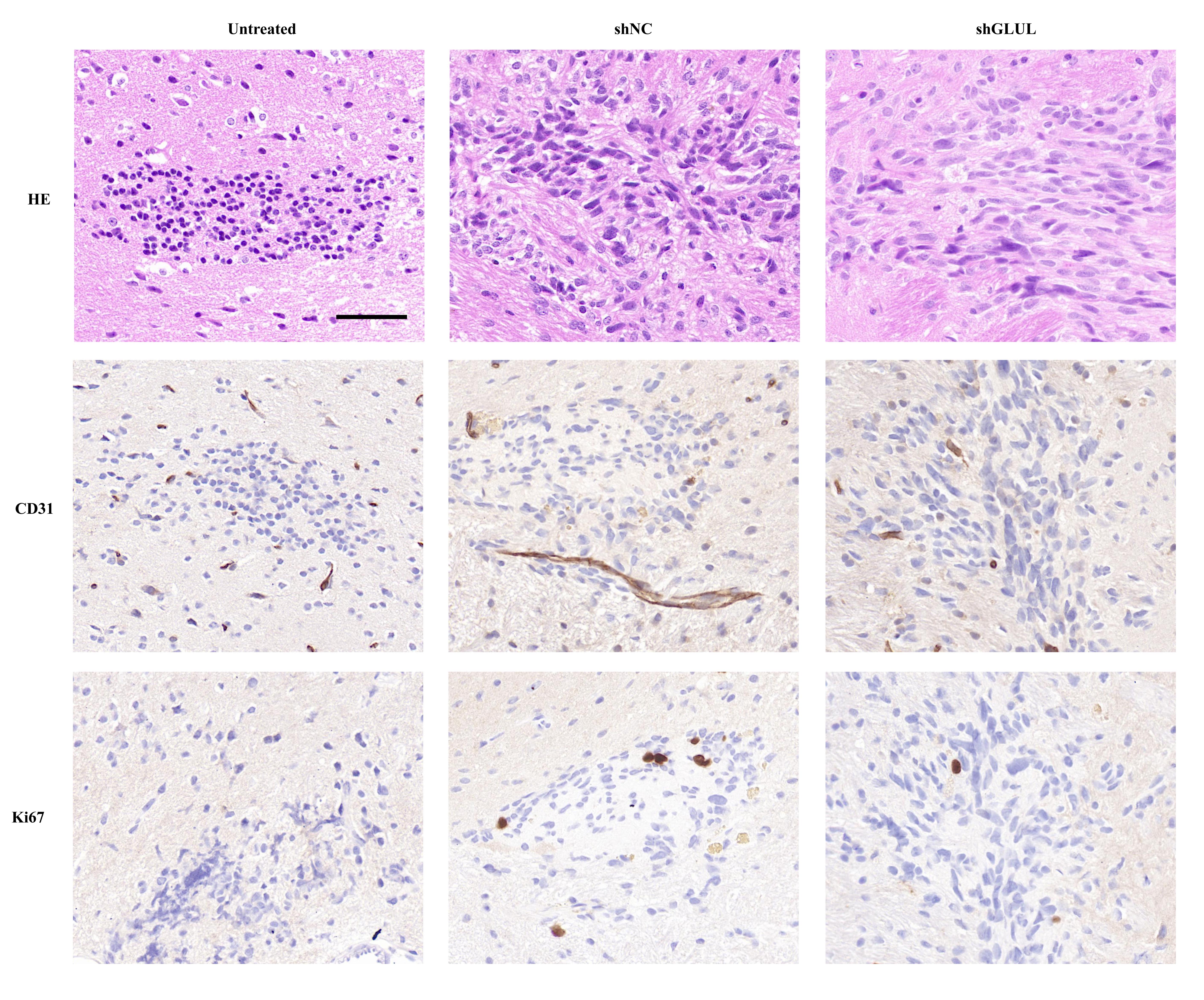
 **Figure S8. Histology staining of orthotopic glioma tissues**. Representative HE images and IHC images stained for CD31 and Ki67. Scale bar, 50 μm.

**Supplementary tables**

**Table S1**. **Sequences of GLUL**

| **NO.** | **Accession** | **Target Seq** | **CDS** | **GC%** |
| --- | --- | --- | --- | --- |
| GLUL-RNAi(85883-1) | NM_002065.7 | CCAGGAGAAGAAGGGTTACTT | 482..1603 | 42.11% |
| GLUL-RNAi(85884-1) | NM_002065.7 | GCACACCTGTAAACGGATAAT | 482..1603 | 36.84% |
| GLUL-RNAi(85885-1) | NM_002065.7 | GCCATGTATATCTGGATCGAT | 482..1603 | 36.84% |

**Table S2**. **Information of primers**

| **Accession** | **Name** | **Sequence（5'-3')** |
| --- | --- | --- |
| NM_002046 | H-GAPDH-S | GGAAGCTTGTCATCAATGGAAATC |
|  | H-GAPDH-A | TGATGACCCTTTTGGCTCCC |
| NM_001033044.4 | H-GLUL-S | AAACGGATAATGGACATGGTGAG |
|  | H-GLUL-A | CTGCTCCCACACCACAGTAATATG |
